# Supplementary material for: Efficient targeting of NY-ESO-1 tumor antigen to human cDC1s by lymphotactin results in cross-presentation and antigen-specific T cell expansion
Source: J Immunother Cancer. 2022 Apr 15;10(4):e004309. doi: 10.1136/jitc-2021-004309 (PMC9014073; doi:10.1136/jitc-2021-004309)
Supplement: Supplementary data [file jitc-2021-004309supp001.pdf]

## SUPPLEMENTARY FIGURES

# EFFICIENT TARGETING OF NY-ESO-1 TUMOR ANTIGEN TO HUMAN cDC1S BY LYMPHOTACTIN RESULTS IN CROSS-PRESENTATION AND ANTIGEN-SPECIFIC T CELL EXPANSION

Camille M. Le Gall[1,2], Anna Cammarata[1], Lukas de Haas[1], Iván Ramos-Tomillero[1,3], Jorge Cuenca-Escalona[1], Kayleigh Schouren[1], Zacharias Wijfjes[1,3], Anouk M.D. Becker[1], Johanna Bödder[1], Yusuf Dölen[1,2], I. Jolanda M. de Vries[1], Carl G. Figdor[1,2,3], Georgina Flórez-Grau[1,*] and M. Verdoes[1,3,*]

1. Department of Tumor Immunology, Radboud Institute for Molecular Life Sciences, Radboudumc, Geert Grooteplein Zuid 28, 6525 GA, Nijmegen, The Netherlands
2. Oncode Institute, Geert Grooteplein Zuid 28, 6525 GA, Nijmegen, The Netherlands
3. Institute for Chemical Immunology, Geert Grooteplein Zuid 28, 6525 GA, Nijmegen, Netherlands.

* corresponding authors: georgina.florezgrau@radboudumc.nl; martijn.verdoes@radboudumc.nl

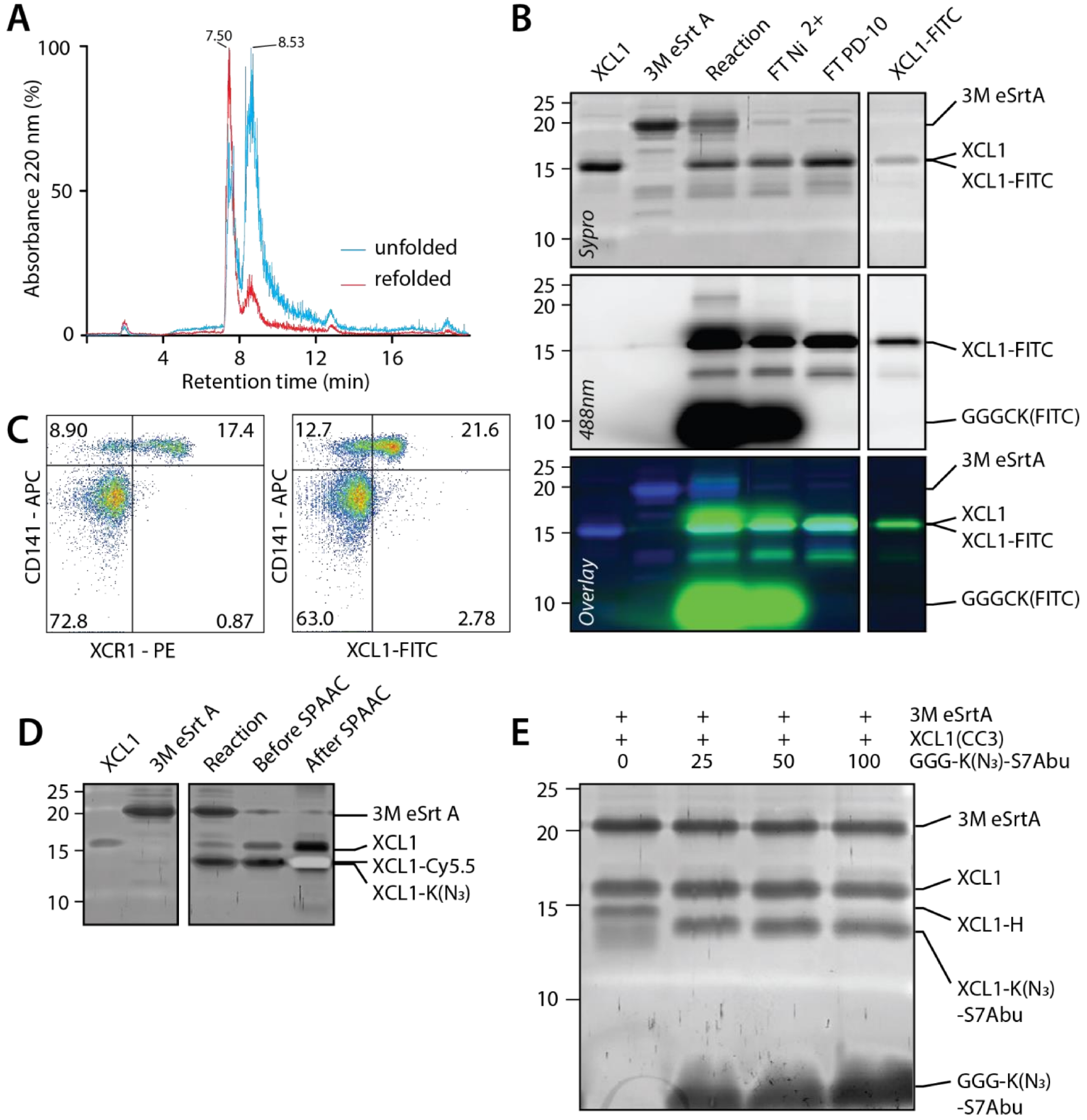


**Figure S1: Production, characterization, and site-specific labeling of XCL1(CC3) with GGG-CK(FITC), GGGK(N3) (with SPAAC to DBCO-C5.5), and GGG-K($N_3$)-S7Abu. A.** HPLC analysis of XCL1(CC3) (refolded, red) and β-ME-treated XCL1(CC3) (unfolded, blue) shows a distinct retention time of XCL1(CC3) upon refolding. Refolding rate was calculated by comparing the ratio of the two species to be over 85%. **B.** Site-specific labeling of XCL1(CC3) (lane 1) with 3M eSrtA (lane 2). After reaction (lane 3), XCL1(CC3)-FITC is purified by nickel affinity purification (FT $Ni^{2+}$, lane 4), FITC excess removed by PD-10 desalting (lane 5) and pure product is obtained after concentration (lane 6). **C.** Commercial anti-XCR1-PE and XCL1(CC3)-FITC identify a comparable subpopulation of ~60% CD141$^{+}$ cDC1s without

staining CD141$^-$ cDC2s, confirming that XCL1(CC3)-FITC specifically binds to XCR1. **D.** Sypro staining in-gel fluorescence. Site-specific labeling of XCL1(CC3) (lane 1) with 3M eSrtA (lane 2) and GGGK($N_3$). After the sortase mediated ligation reaction (lane 3), XCL1(CC3)-K($N_3$) is purified by PD-10 desalting to remove the excess peptide. The flow-through (lane 4) is subjected to SPAAC (lane 5) with DBCO-Cy5.5 leading to XCL1-Cy5.5 (Sypro fluorescence is absorbed by Cy5.5). **E.** Sypro staining in-gel fluorescence. Small-scale optimization of GGG-K($N_3$)-S7Abu peptide equivalents for site-specific labeling of XCL1(CC3) shows that only 25 equivalents of GGG-K($N_3$)-S7Abu are necessary to generate XCL1(CC3)-K($N_3$)-S7Abu with minimal hydrolysis byproduct (XCL1-H).

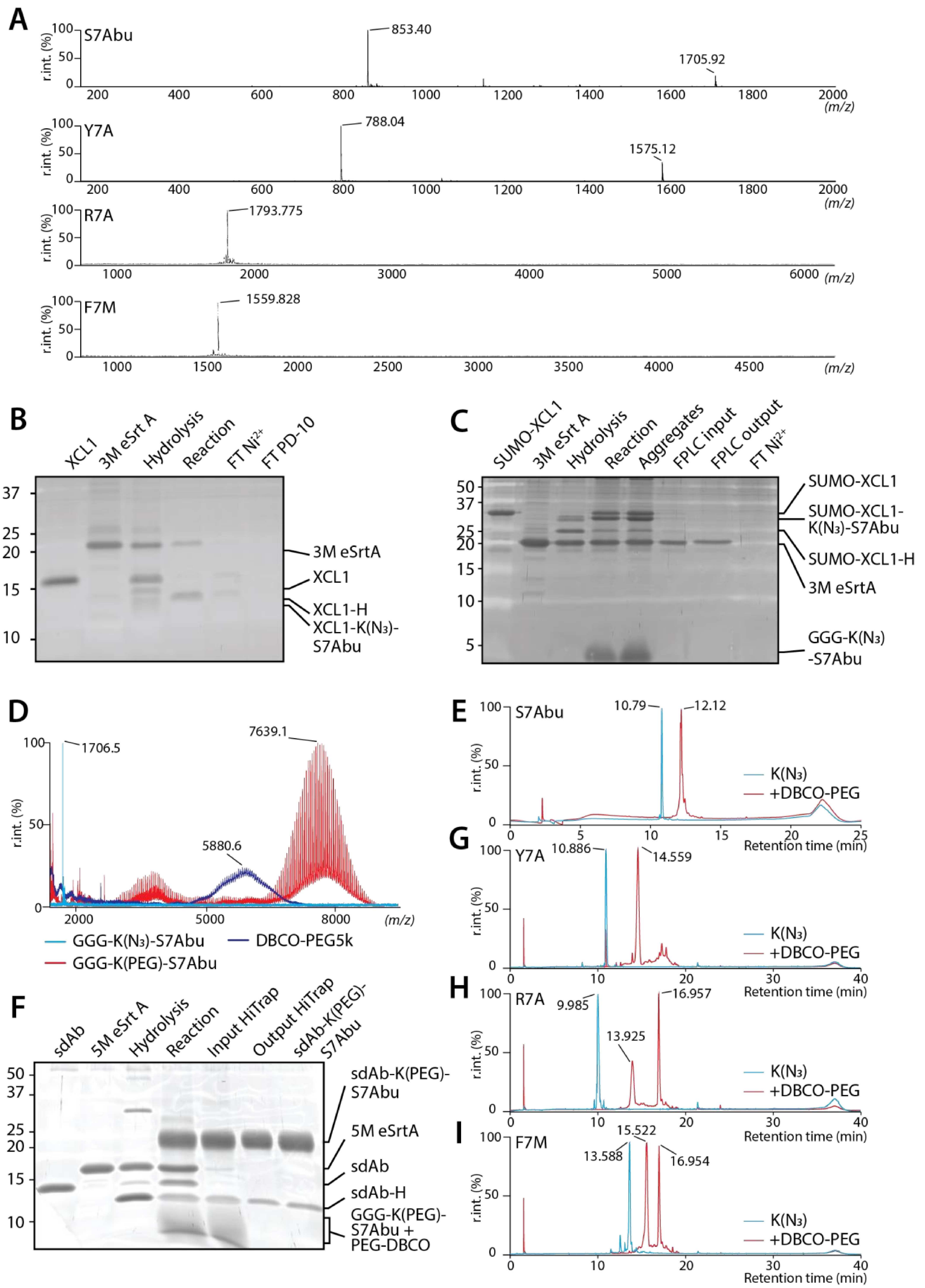
A
S7Abu
853.40
1705.92
Y7A
788.04
1575.12
R7A
1793.775
F7M
1559.828
r.int. (%)
(m/z)
B
XCL1
3M eSrt A
Hydrolysis
Reaction
FT Ni2+
FT PD-10
3M eSrtA
XCL1
XCL1-H
XCL1-K(N3)-S7Abu
C
SUMO-XCL1
3M eSrt A
Hydrolysis
Reaction
Aggregates
FPLC input
FPLC output
FT Ni2+
SUMO-XCL1
SUMO-XCL1-K(N3)-S7Abu
SUMO-XCL1-H
3M eSrtA
GGG-K(N3)-S7Abu
D
1706.5
7639.1
5880.6
GGG-K(N3)-S7Abu
DBCO-PEG5k
GGG-K(PEG)-S7Abu
E
S7Abu
10.79
12.12
K(N3)
+DBCO-PEG
Retention time (min)
G
Y7A
10.886
14.559
H
R7A
9.985
13.925
16.957
I
F7M
13.588
15.522
16.954
F
sdAb
5M eSrt A
Hydrolysis
Reaction
Input HiTrap
Output HiTrap
sdAb-K(PEG)-S7Abu
sdAb-K(PEG)-S7Abu
5M eSrtA
sdAb
sdAb-H
GGG-K(PEG)-S7Abu + PEG-DBCO

**Figure S2: PEG-ylation of K($N_3$)-S7Abu, K($N_3$)-Y7A, K($N_3$)-R7A and K($N_3$)-F7M enables purification of stable XCL1(CC3)-K(PEG)-antigen conjugates. A.** MS analysis of GGG-K($N_3$)-S7Abu, GGG-K($N_3$)-Y7A, GGG-K($N_3$)-R7A and GGG-K($N_3$)-F7M used in this study. **B.** Site-specific labeling of XCL1(CC3) (lane 1) with GGG-K($N_3$)-S7Abu allows XCL1(CC3)-K($N_3$)-S7Abu product formation (lane 4), but product could not be purified (lanes 5 and 6). **C.** Site-specific labeling of SUMO-XCL1 (XCL1 fused to its solubility domain) with GGG-K($N_3$)-S7Abu allows product formation (lane 4), but product is present in aggregates formed during reaction (lane 5) and could not be isolated (lanes 6-8). **D.** MALDI-TOF analysis of GGG-K($N_3$)-S7Abu, DBCO-PEG5k and GGG-K(PEG)-S7Abu shows an increase in *(m/z)* upon PEGylation, and reaction completion. **E.** HPLC analysis of GGG-K($N_3$)-S7Abu before and after PEGylation shows a distinct retention time, and reaction completion. **F.** sdAb-K(PEG)-S7Abu can be generated and purified by cation exchange, with minimal hydrolysis product present. Densitometry was performed to calculate the concentration of sdAb-K(PEG)-S7Abu used in cell experiments. **G, H and I.** HPLC analysis of GGG-K($N_3$)-Y7A (G), GGG-K($N_3$)-R7A (H) and GGG-K($N_3$)-F7M (I) before and after PEGylation shows a distinct retention time, and reaction completion.

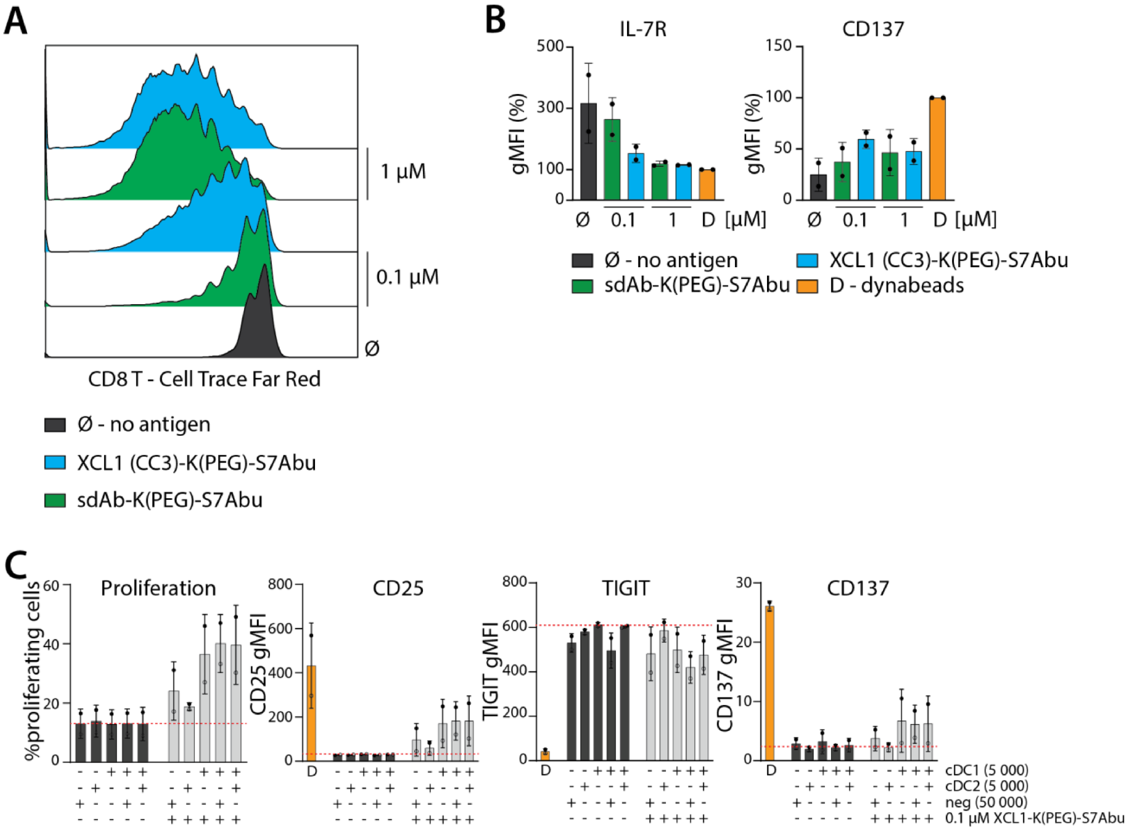


**Figure S3: XCL1(CC3)-K(PEG)-S7Abu efficiently targets cDC1s, and is degraded to present S7Abu CD8$^+$ T cells. A.** CD8$^+$ T cell proliferation tracked by cell trace dye upon treatment of cDC1s with XCL1(CC3)-K(PEG)-S7Abu or sdAb-K(PEG)-S7Abu shows the selective advantage of XCR1 targeting. Representative of N=3 independent donors. **B.** IL7R and CD137 expression on activated CD8$^+$ T cells. N=2 independent donors. **C.** cDC1s and cDC2s were purified from CD14$^-$CD3$^-$CD56$^-$CD19$^-$ enriched PBMCs of HLA-A*02:01$^+$ donors. The negative fraction following cDC1 and cDC2 isolation (CD3$^-$CD14$^-$CD56$^-$CD19$^-$CD1c$^-$CD141$^-$) was kept. $5\times10^3$ purified cDC1s, $5\times10^3$ purified cDC2s or $5\times10^4$ cells from the negative fraction (neg) were pulsed with 0.1 µM XCL1-K(PEG)-S7Abu for 3 h and incubated with 50 000 CD8$^+$ T cells. Purified cDC2s were not able to efficiently prime CD8$^+$ T cells compared to cDC1s, and cDC1s were able to activate and induce comparable proliferation of CD8$^+$ T cells in presence or absence of an excess of contaminating cells (neg). These results show that XCR1 targeting on cDC1s induces CD8$^+$ T cell activation despite the presence of other cells. D: Dynabeads. N=2 independent donors.
